# A randomized controlled trial comparing conventional and piezosurgery methods in mandibular bone block harvesting from the retromolar region

**DOI:** 10.1186/s12903-023-03739-9

**Published:** 2023-12-09

**Authors:** Ferit Bayram, Ahmet Demirci

**Affiliations:** Department of Oral and Maxillofacial Surgery, Faculty of Dentistry, Marmara University, Basibuyuk Yolu 9/3, Basibuyuk, Maltepe, Istanbul, 34854 Türkiye

**Keywords:** Alveolar ridge augmentation, Bone transplantation, Clinical trial, Dental implantation, Piezosurgery

## Abstract

**Background:**

Although piezosurgery is now commonly used for various applications in maxillofacial surgery, its advantages over conventional rotary instruments in terms of postoperative edema, ecchymosis, postoperative morbidity, and prolonged osteotomy time have been questioned.

**Materials and methods:**

This study aimed to compare the efficiency, postoperative morbidity, and complication rates of piezosurgery and conventional methods in harvesting autogenous ramus grafts. In this randomized controlled trial, 21 patients (32 sides) underwent autogenous graft harvesting from the ramus area, with 16 sites treated using piezosurgery and 16 using the conventional method. The primary outcomes measured were osteotomy time, total operation time, and postoperative morbidity. Complication rates were also evaluated.

**Results:**

The final analysis encompassed 19 patients, accounting for a total of 30 donor sites, following the exclusion of two patients who were unable to attend the scheduled follow-up visits. A total of 19 patients (30 donor sites) were included in the final analysis. No statistically significant difference was found in the mean osteotomy time between the piezosurgery group (mean: 10.35, SD: 2.74 min) and the conventional group (mean: 8.74, SD: 2.74 min) (95% CI: -3.67 to 0.442, p = 0.119). The total operation time, postoperative pain, and swelling were not significantly different between the two groups (p > 0.05). The complication rates, including wound dehiscence and inferior alveolar nerve exposure, were similar in both groups.

**Conclusions:**

Piezosurgery can be safely used for harvesting autogenous ramus grafts and does not increase osteotomy or total operation time compared to the conventional method. The postoperative morbidity and complication rates were also similar, indicating that both techniques can be effectively employed in clinical practice.

**Clinical Trial Registration:**

The protocol was registered on clinicaltrials.gov (ID: NCT05548049, First registration date: 21/09/2022).

## Introduction

The basic principle of osseointegration, as defined by Branemark, is based on establishing a structural and functional connection between the living bone and the implant surface [1]. In cases where there is not enough bone, the alveolar defects in the jaws need to be repaired to establish this connection. Despite all the advances in biomaterials and their expanding use, autogenous grafts are still the gold standard for treating significant defects in the jaws [2]. Different donor sites have been defined for autogenous graft harvesting, including intraoral and extraoral sites [3]. The donor site is selected by assessing the following factors: graft quality, graft quantity and both patient and surgeon preference. The retromolar ramus is one of the most preferred donor sites by patients and surgeons because of its high survival and success rate, low surgical invasiveness, and minimal patient concern for altered facial contour [4]. The most significant drawback of autogenous grafts is donor site morbidity, which is more critical because of the nerves and vessels in the retromolar region [5].

Conventionally, fissure and round burs were used to harvest grafts from the retromolar region. However, the use of diamond discs, microsaws and piezosurgery [6–8] has increased. Among these methods, piezosurgery is one of the most well-known methods of reducing donor site morbidity [9]. Piezosurgery is increasingly used in different branches, such as orthopedics, ENT, neurosurgery and dentistry because it is selective for mineralized tissues and does not damage adjacent soft tissues such as nerves and vessels [10]. Additionally, in the field of implantology, piezosurgery is also being utilized, with emerging research indicating its efficacy. Comparative studies between osseodensification drills and piezoelectric implant site preparation have yielded similar results in terms of outcomes [11]. The most crucial disadvantage of this method is the inconclusive results in the literature related to different surgical procedures, which may prolong the operation time and cost. While many similar high-quality studies show that piezosurgery may be slower when performing wisdom tooth extraction [12], inconsistent findings suggest that it may shorten or slightly prolong the operation time in operations such as sagittal splitting, sinus lifting, or implant socket preparation [13, 14]. Based on the osteotomy time, the anatomy of regions such as the retromolar area and access to the region may be affected by factors such as using the appropriate tip to access the site and the surgeon’s experience with the device [15]. In addition, the region has a complex anatomy; by using a device that minimizes nerve damage, there may be differences in the operation time compared to the conventional method.

Due to these different results in the literature, it is difficult to conduct a meta-analysis comparing piezosurgery with the conventional method in an important issue such as donor site morbidity. Since the major criticism about piezosurgery is the slowness of the device, in this randomized controlled study, we aimed to compare the piezosurgery method with the conventional rotary method in autogenous graft harvesting from the retromolar ramus focusing on the osteotomy time. In this study, our primary aim was to test the null hypothesis that there is no difference in osteotomy times between the groups. Our secondary aims were to statistically compare pain, trismus, and edema and to report possible postoperative complications.

## Method and materials

### Study design

This study’s findings are presented following the CONSORT (Consolidated Standards of Reporting Trials) guidelines. The study protocol was approved by the Marmara University Faculty of Medicine Clinical Research Ethics Committee (approval no: 09.2021.725). The sample size estimation for this study was based on the number of donor sites rather than the number of patients. This approach was chosen because each patient could have more than one donor site, and our primary focus was on comparing the outcomes between the different surgical techniques at the level of individual donor sites. The sample size calculation was performed based on the primary outcome, which was the difference in osteotomy times between the piezosurgery and conventional methods. The calculation was performed using G*Power software, considering a significance level (alpha) of 0.05 and a power (1-beta) of 0.8. The effect size was determined from previous literature on similar procedures [16]. To strengthen the study’s findings, we decided to raise the sample size by 10%. This randomized controlled trial, consisting of conventional and piezosurgery groups (allocation ratio = 1:1), was conducted between September 2021 and March 2022 at Marmara University Faculty of Dentistry, Department of Oral and Maxillofacial Surgery. The trial did not employ stratification in patient allocation, focusing instead on a broadly representative sample without distinction based on age, gender, or comorbidities. All consecutive unrelated patients who presented to the clinic between these dates with the complaint of single or multiple missing teeth but who did not have sufficient horizontal bone for implants were invited to participate in the study. The preoperative analysis of the participants included obtaining a complete medical history, clinical and radiographic examination of the oral and maxillofacial region, and comprehensive analysis of the donor and recipient sites. The main inclusion criteria were severe alveolar ridge atrophy in the horizontal plane (≤ 4 mm) and no accompanying vertical defects according to preoperative cone-beam computed tomography scans (Morita Veraview IC5, Kyoto, Japan). Donor site selection for defect repair was determined by the defect morphology and recipient site location. Inclusion and exclusion criteria were determined as shown in (Table 1). The treatment process was explained to all patients, and written informed consent was obtained from patients who agreed to participate in the study. The protocol prepared before the start of the study was not modified until the end of the study.

**Table 1:** Inclusion and exclusion criteria

**Inclusion Criteria** One or two missing teeth In the CBCT measurement, a patient with sufficient bone vertically (> 7 mm) but < 4 mm bone horizontally and who will undergo lateral augmentation with autogenous block bone No neurosensory disturbance in the IAN or lingual nerve Systemically healthy ASA I-II Bone graft in the donor site (ramus) with sufficient volume for the recipient site **Exclusion Criteria** Patients with general contraindications to implant surgery Patients with cleft lip and palate and defects exceeding the alveolus, as their baseline clinical status was not comparable to the study group Patients with defects caused by tumors or osteoradionecrosis Presence of acute or chronic infection at the recipient site Smoking > 10 cigarettes per day Uncontrolled diabetes, past or current use of antiresorptive or antiangiogenic drugs Patients who had COVID after the graft operation until implant surgery or who did not attend follow-up appointments were excluded from the study.	

*Note*: Abbreviations: IAN, inferior alveolar nerve; ASA, American Society of Anesthesiologists

For the random allocation of participants in our study, we utilized a computer-generated list of random numbers in two blocks (Excel, Microsoft Corp, Redmond, WA, USA), assigning participants to either the conventional or piezosurgery groups. Each new patient received a unique number from this list. Corresponding sealed, opaque envelopes were prepared, with their contents unknown to both participants and researchers. These envelopes were only opened after fulfilling the needed criteria and obtaining participant consent. Due to the inherent requirements of the surgical procedures, the physician was necessarily informed of the group allocation to perform the appropriate surgical technique. However, to maintain the integrity of the study and minimize potential bias, the investigator responsible for data collection and analysis was blinded to the technique used. This individual, who had access to medical data, was not involved in the surgical procedures and was unaware of the group assignments.

### Surgical procedure

**Fig. 1:**
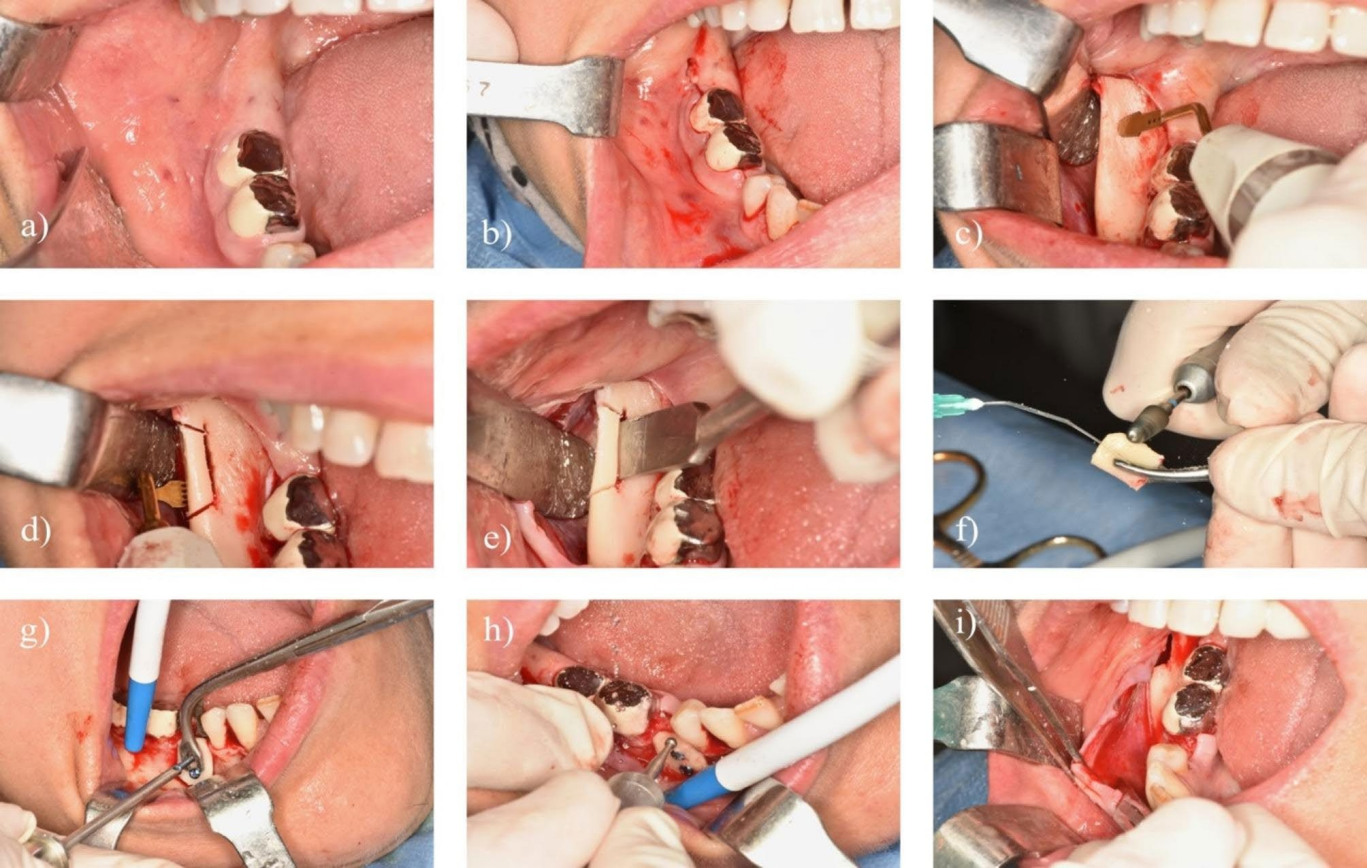
Clinical picture of surgical procedure of bone harvesting from retromolar area **a**) baseline situation, **b**) incision similar to wisdom tooth extraction, **c**) osteotomy with piezosurgery tip OT-12, **d**) inferior osteotomy with tip OT8-R, **e**) bone block separation with a chisel, **f**) modifying the harvested graft, **g**) adaptation of graft to the recipient site, **h**) final adaptation of graft, **i**) periosteal releasing incision for tension-free closure of the flap

All procedures were performed under local anesthesia and standardized as much as possible. Oral hygiene education was given to all patients. Patients were asked to rinse with chlorhexidine 0.2% chlorhexidine three times a day, starting from the day after surgery to the day of suture removal. Mouth washing with 0.2% chlorhexidine solution was also performed preoperatively. A local anesthetic containing a vasoconstrictor (articaine hydrochloride) was infiltrated into the retromolar region, ascending ramus, and masseter area to block cervical innervation. When the donor and recipient sites were on the same side, the incision was made like the wisdom tooth incision, extended as distally as necessary, and the full-thickness flap was removed. When the recipient and donor sites were in different quadrants, the incision was similar to the sagittal split incision. The masseter was reflected laterally using the Minnesota retractor. Four osteotomies were performed as described by Misch (Fig. 1) [17]; (1) an external oblique cut was performed 4–6 mm medial to the external oblique ridge, and the length of the osteotomy was determined by the size of the area to be grafted (2); superior ramus cut: perpendicular to the external oblique cut in the lateral cortex of the ramus (3), inferior ramus cut: this osteotomy included only the cortex and was made parallel to the external oblique cut (4), anterior body cut: since the position of the mandibular canal is the most lateral at the wisdom tooth level, the vertical osteotomy was made at the level of the second or first molar, depending on the need for the graft, and the osteotomy was deepened until the spongious bone and hemorrhage were visible.

### Conventional group

Cortical incisions were made under sterile saline irrigation and maintained at a cooled temperature, for both the experimental and conventional groups. This procedure involved using a thin #9 fissure bur (HM 31 009, Hager & Meisinger, Neuss, Germany) and a surgical handpiece S-11 Straight tip (W&H Dentalwerk, Bürmoos, Austria) at 20,000 rpm. The physiodispenser settings (NSK Surgic Ap, Tokyo, Japan) adhered to the manufacturer’s recommendations. New round and fissure burs were used for each patient to ensure sterility and consistency.

### Piezosurgery group

During the use of the piezosurgery device (Mectron s.p.a., Carasco, Italy), a saline solution stored at 4 °C was used to increase the cooling effect, and 1-minute breaks were given for the handpiece to cool down for more than 5 min. The sound of the cut was used as acoustic feedback to ensure that the handpiece and tips were not pressed down too much, thus affecting the cutting efficiency and temperature of the bone. The device was used in boosted mode with high-frequency vibration for optimum bone-cutting capacity. Laser markings on the tips were used to control the depth of the incision. Superior, anterior body and external oblique incisions were made with OT-12, while the inferior ramus cut was made using OT8-L or OT8-R tips, depending on the studied quadrant (Mectron s.p.a., Carasco, Italy). The same tip was used up to 3 times if it was not broken before (Fig. 1).

After the osteotomies were completed in both groups, the block graft was carefully separated from the donor site and stored in 0.9% saline solution. The bone obtained was used for horizontal augmentation of the bone defect in the mandible and maxilla.

A full-thickness flap with a unilateral vertical incision accompanied by a sulcular incision at the recipient site was removed, and the bone defect was exposed. The length of the screw was decided using a caliper to measure the width of the recipient site. Fixing the graft to the recipient site was performed with one or two 1.6 mm microscrews. Any sharp edges were smoothed using a round bur. Maximum bone-to-bone contact between the harvested and native bone was attempted. No decortication was performed, and no membrane was applied. A periosteal-releasing incision was made for tension-free closure of the graft in the recipient site (Fig. 1). The flap was closed with resorbable simple sutures.

All patients were prescribed paracetamol for postoperative pain control and antibiotics for seven days, starting one day before surgery and administered every 12 h. A cold compress for 48 h was recommended. Sutures were removed after ten days.

The flap design for the second surgery at 4 months (re-entry surgery) was similar to that of the first surgery, but the releasing incision in the vestibule was shorter. Screws fixing the bone block were removed, and dental implants (Straumann AG, Basel, Switzerland or Megagen, Seoul, South Korea) were placed according to the patient’s preference and the standard surgical protocol.

### Outcome measures

The primary outcome measure of the study was to the compare osteotomy time between the conventional method and piezosurgery. In addition, flap elevation, graft adaptation time and total operation time were recorded using a stopwatch on the watch application on the iPad. The lap button was pressed when each stage was passed. The total operation time was recorded from the first incision to the last suture. Osteotomy time was the time from full-thickness flap removal to complete separation of the block graft from the donor site. Adaptation time was also recorded. This focus on osteotomy time as the primary outcome was selected to directly address and quantify the primary disadvantage associated with piezosurgery in the context of oral and maxillofacial surgical procedures.

Postoperative pain was recorded using the VAS scale. The patients were asked to score the pain in the relevant region using a visual analog scale, between 1 and 10 points according to the severity on the first day, third day, seventh day, and 14th day after surgery. In addition, patients were asked to record the number of analgesics used in the first week [18].

Trismus was evaluated by measuring the distance between the mesio-incisal corners of the upper and lower central incisors in millimeters, using a ruler, at maximum mouth opening. In our study, postoperative edema was assessed using the technique outlined by Neupert et al., which involves linear measurements with a tape measure. This approach entailed taking measurements from five specific points: (1) from the mandibular corner to the tragus (Go-Tr), (2) from the mandibular corner to the lateral canthus of the eye (Go-Ca), (3) from the mandibular corner to the nose wing (Go-An), (4) from the mandibular corner to the oral commissures (Go-Cm), and (5) from the mandibular corner to the pogonion (Go-Pog). We acknowledge the emergence of new three-dimensional assessment techniques for facial swelling, particularly in split-mouth studies, as highlighted in recent publications [19].

Piezo tip or bur fracture, osteosynthesis screw fracture, and nerve exposure were recorded as intraoperative complications, and mucosal dehiscence, infection and neurosensory disturbance were recorded as postoperative complications. A two-point discrimination test with calipers was used for postoperative neurosensory disturbance evaluation. Patients were asked to distinguish between the number of contacts on the oral mucosa and skin while their eyes were closed. If two points could be distinguished at less than 7 mm, it was considered normal; at a distance between 7 and 11 mm, it was considered slightly aberrant; and if it could only be distinguished at a distance greater than 11 mm, sensitivity was considered impaired [32]. The graft was considered successful when there was no infection or graft loss. Sufficient bone volume was obtained to allow implant placement.

### Statistical analysis

For descriptive statistics, mean and standard deviation, median and minimum and maximum, frequency, and ratio values are presented. The distribution of variables was measured using the Kolmogorov–Smirnov test. Independent sample t tests and Mann–Whitney U tests were used for comparing operation and osteotomy times; Mann–Whitney U and Wilcoxon tests were used for comparing postoperative pain, edema and swelling parameters, and chi-squared tests were used for the analysis of qualitative independent data. SPSS v28.0 software (IBM SPSS Inc., Armonk, NY, USA) was used for the analysis. Intraoperative and postoperative complications were only recorded and not included in the intergroup analysis. This study analyzed data based on the actual treatments received by the participants, without applying an intention-to-treat approach.

## Results

**Fig. 2:**
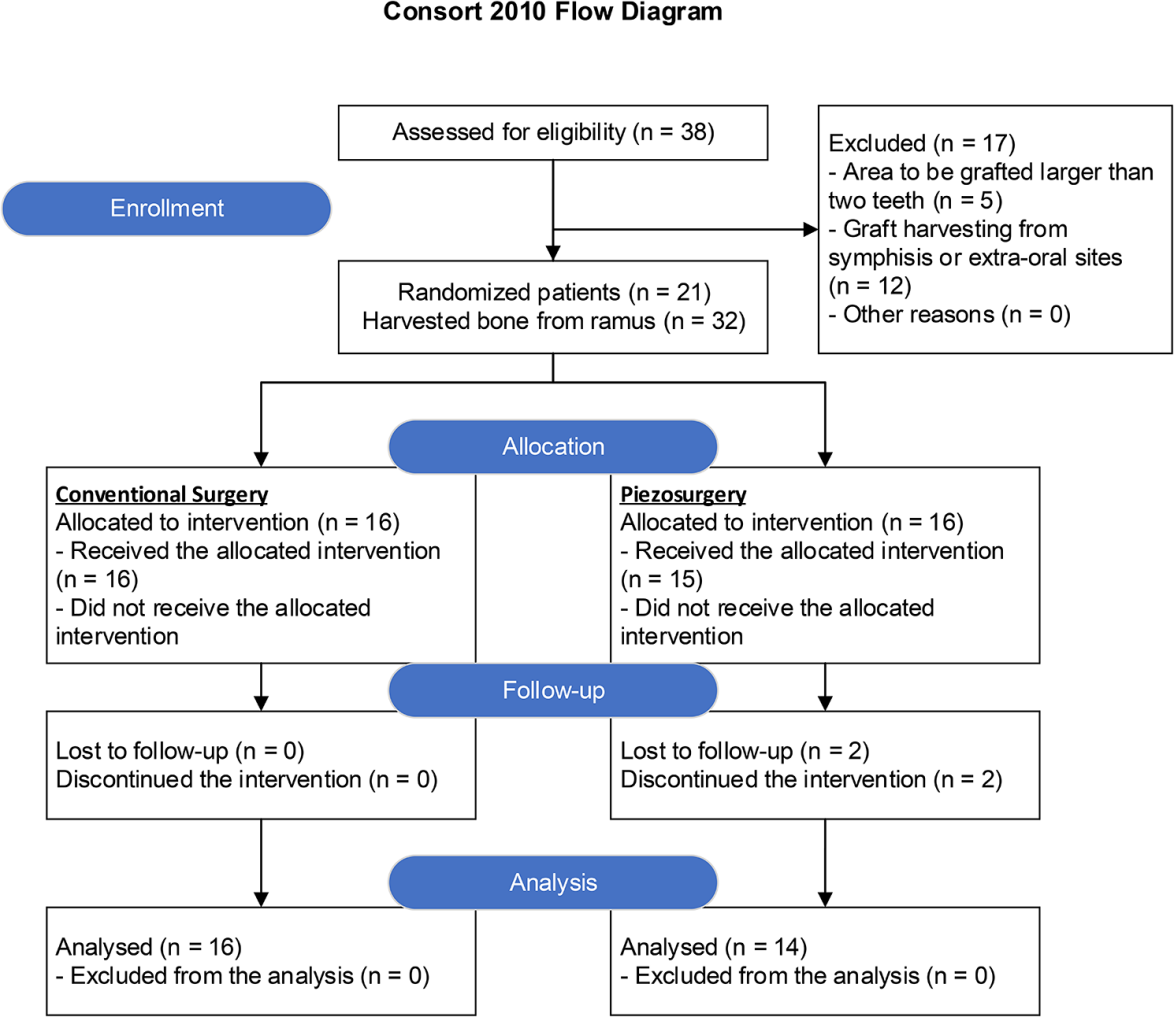
CONSORT 2010 flow diagram

A total of 19 patients with 30 donor sites were included in the study, with 16 donor sites in the conventional group and 14 donor sites in the piezosurgery group (Fig. 2). The demographic and clinical characteristics of the patients in both groups were similar, with no significant differences observed (p > 0.05) (Table 2). Two patients were excluded from the study due to their lack of attendance at follow-up visits. These patients had baseline data, but they failed to attend the scheduled follow-up appointments, making the assessment of postoperative outcomes impossible. The final analysis was based on a per-protocol approach, including only the 19 patients who completed the study, with drop-outs and missing data excluded from the analysis.

**Table 2:** Characteristics of included patients

	Conventional Surgery	Piezosurgery
Gender (female/male)	10/4	10/5
Age at the time of surgery, mean (SD)	50 ± 11.3	46.3 ± 13.04
Smoking habits	2	2
Initial alveolar bone thickness mean (SD)	2.91 ± 0.75	3.14 ± 1.00
The resulting alveolar bone thickness mean (SD)	5.57 ± 1.01	5.75 ± 1.16

**Fig. 3:**
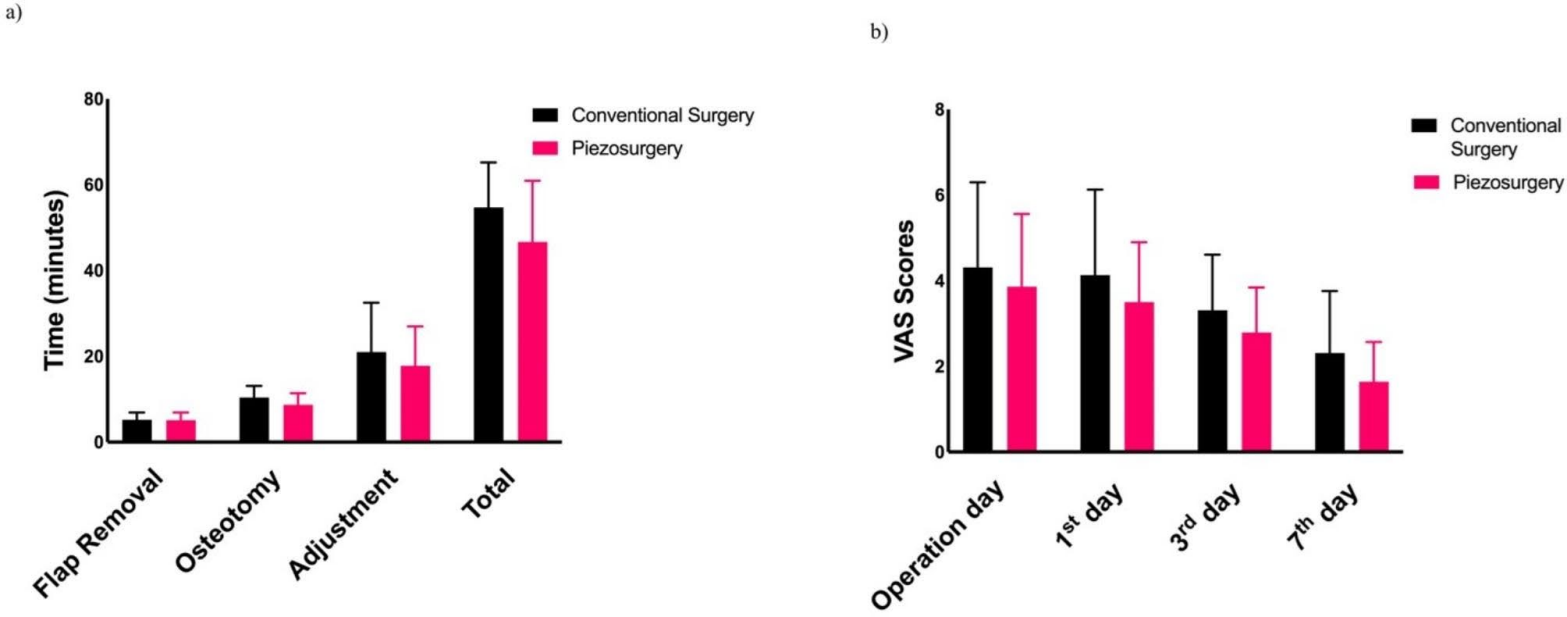
Comparison of operation time and postoperative pain scores between groups

Although the mean osteotomy time was lower in the piezosurgery group, it was not statistically significant (95% CI: -3.67 to 0.442, p > 0.05p = 0.119). Although there was a minor difference between the groups in flap lifting, graft adaptation (95% CI: -11 to 4.70, p = 0.418) and total osteotomy times (95% CI: -14.4 to 2.20, p > 0.05p = 0.090) were also lower in the piezosurgery group (Fig. 3). Nevertheless, there was no statistically significant difference between the groups; therefore, the null hypothesis was not rejected.

Pain was compared on the operation day, postoperative day 1, postoperative day 3, and postoperative day 7. There was no significant difference in the VAS score between the conventional and piezosurgery groups (*p* > 0.05). In within-group analyses, there was no significant difference between the operation day VAS score and the postoperative third-day VAS score in the conventional group, while these values differed significantly in the piezosurgery group (*p = 0.049*) (Fig. 3). The average total number of analgesics taken during the first week after surgery was not significantly different between the two groups (*p > 0.05*).

**Fig. 4:**
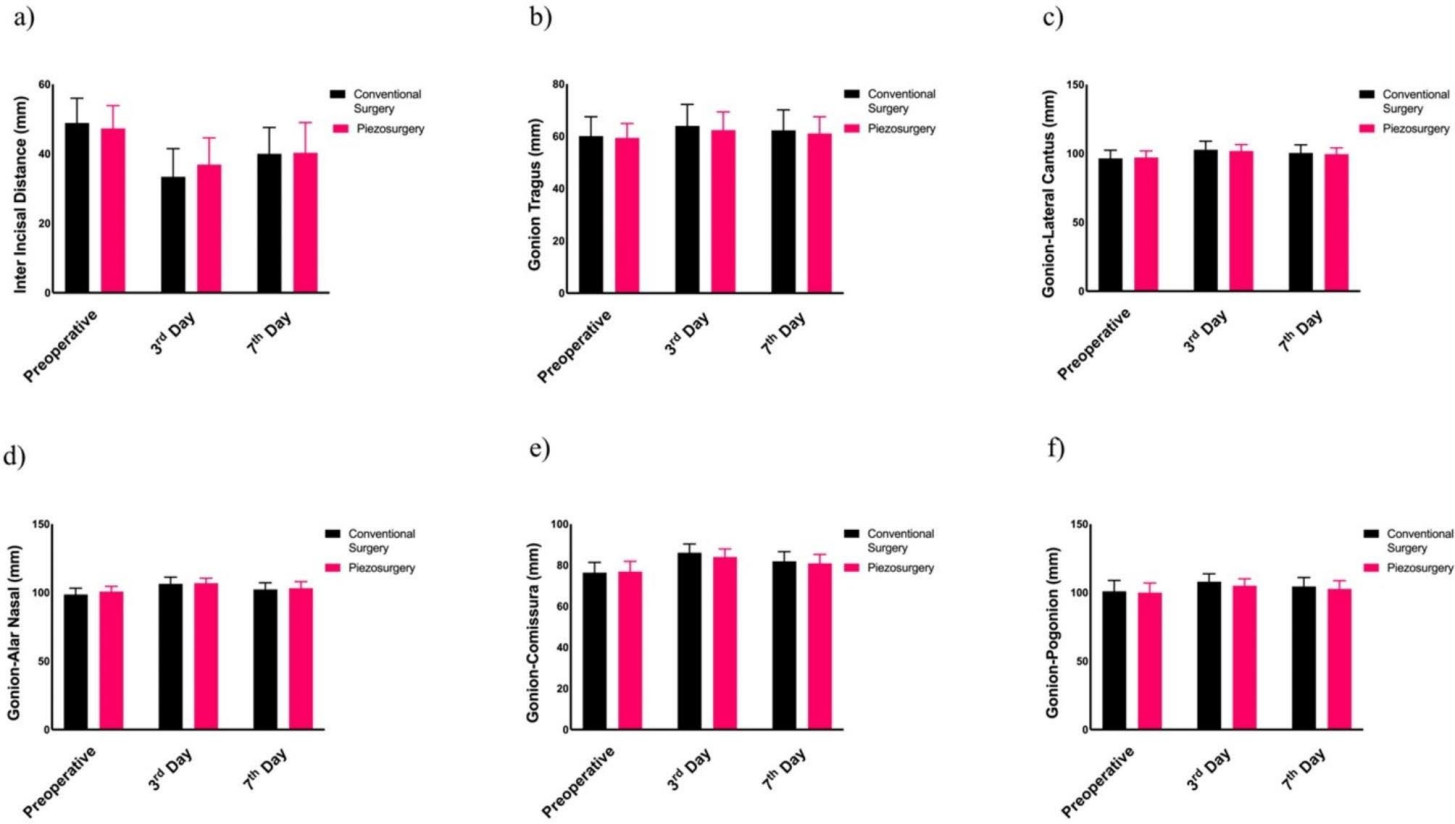
Comparison of postoperative trismus and edema values between groups

When the patients’ postoperative interincisal mouth opening measurements were analyzed, there was no significant difference between the preoperative and the seventh day values (*p* > 0.05). However, there was a significant difference between the preoperative and the postoperative third day values between the conventional and piezosurgery groups (*p* < 0.05) (Fig. 4).

When the swelling measurements of the five points were evaluated in the patients, there was no significant difference between the conventional and piezosurgery groups in terms of the increase in Go–Tr, Go–Ca, Go–An, and Go–Pog values from preoperatively to the third day, although there was a significant difference in the Go–Cm values *(p = 0.03*). When the increase from preoperative to the seventh postoperative day was examined, there was no significant difference between the conventional and piezosurgery groups in terms of the percentage increase in the Go–Tr, Go–Ca, Go–An, Go–Cm, and Go–Pog values (Fig. 4).

## Complications

In three grafting sites, wound dehiscence was observed in the early postoperative period. In this case, mouthwash was used, and the sharp edges of the exposed graft were corrected with a round bur under saline irrigation. Patients were followed up until implant placement surgery with regression of dehiscence. In one patient, the bone block separated from the residual bone during the implant placement session. In this case, implant placement surgery was performed simultaneously with directed tissue regeneration. In five surgical sites, the mandibular nerve was exposed during block grafting. All these patients reported mild numbness of the lips at follow-up one week later. This transient numbness lasted eight weeks, and none of the patients had numbness during the implant placement session. A total of 46 implants were placed in 16 patients (4 Megagen Anyone, Megagen, Seoul, South Korea, 2 Strauman Bone Level, Straumann AG, Basel, Switzerland and 40 Megagen ST, Megagen, Seoul, South Korea). No significant differences were found between the two groups regarding the number of complications (p > 0.05). The multilevel mixed-effects model analysis confirmed the robustness of these findings, taking into account the within-patient correlations.

## Discussion

Every attempt to reduce the associated morbidity when deciding on the donor site is essential in determining the frequency of clinical use of a method. One of the ways to reduce morbidity is to be gentle with the tissues and to keep the operation time short. In this study, conventional and piezosurgery methods were compared in autogenous graft harvesting from the ramus, which is one of the most preferred donor sites when there is sufficient bone. In this randomized controlled study, no significant difference was found in osteotomy times between the groups. Although there was a substantial increase in operation time compared to the conventional method in operations such as separation of dental hard tissue with bone that would be performed in wisdom tooth surgery, it was found that piezosurgery does not work slowly due to its other advantages, especially in bone-related operations involving factors other than just cutting the bone such as bone harvesting from the retromolar area.

The disadvantages of the piezosurgery device have been widely discussed in the literature; slowing the osteotomy time by 20–35% and increased costs are the most debated disadvantages [20]. Of these disadvantages, the incremental cost is not the subject of our study, and no calculation has been made. Regarding the duration of the operation, different results have been reported for other procedures, and it is challenging to make a definitive judgment. A meta-analysis of randomized controlled trials, which came closest to a consensus for wisdom tooth surgery, reported that it prolonged the duration and decreased postoperative pain and edema in impacted tooth surgery [21]. In another meta-analysis, piezosurgery was found to prolong the operation time in lateral sinus floor augmentation [22]. In a randomized controlled study in which autogenous grafts were taken from the retromolar region with piezosurgery as a comparison group with microsaw, the graft harvest time was 16.47 ± 2.74 [7]. We think that this time is different from our study because we took smaller volumes of graft, and there was no difference between the groups because the different tips used may have shortened the time.

Different tips have been developed for piezoelectric devices, which can provide significant advantages with safe and effective osteotomies [23]. Different tip options can provide a better cutting effect when the tip is transformed into an electric micrometer under the influence of ultrasonic vibrations [23]. Previous studies have shown that piezoelectric ultrasonic equipment allows more precise cuts than rotary handpieces [24]. This is particularly important in regions such as the ramus, which are relatively difficult to access. In this respect, piezosurgery is more precise and safer because it requires the active tip to be used at low amplitude in a small area. Considerable time savings efforts can be achieved using different tips. In most comparative studies, the working settings of the piezosurgery device and the tips used were not mentioned. In an in vivo study, incisions made with an OT12S showed a cut surface highly similar to the original bone morphology, with tiny debris remaining, a low depth of cut, and a low bone carbonization [25].

When comparing piezosurgery with the conventional method, it is difficult to reach a conclusion about edema and pain in oral and maxillofacial surgical procedures. Although many studies claim that there is no significant difference between piezosurgery devices and rotary instruments [26], other studies have shown that they significantly reduce postoperative sequelae [27]. Our study found no significant difference between the groups in edema and pain VAS scores. However, since we placed no restrictions on the painkillers and anti-inflammatories that patients used postoperatively, these results should be carefully considered. No study has investigated whether pain and swelling were low due to the number of painkillers used, and it is impossible to establish a causal relationship between them. The results of these studies were consistent with the idea that the lateral bone block augmentation procedure showed very low postoperative morbidity when a standard pain management protocol was followed [28].

Clinical and animal studies have shown that piezosurgery is gentler than the conventional method of bone and soft tissue during osteotomy [29]. Because the device only works on mineralized tissues, it protects vital soft tissues, including the mucosa, nerves, and blood vessels. Piezosurgery is suitable for collecting bone particles of ideal size and generates low heat, thus minimizing the possibility of thermal necrosis [8]. However, piezosurgery is not entirely harmless to soft tissues, and piezoelectric vibrations can cause long-term soft tissue trauma. Inferior alveolar nerve paresthesia is the most common type of morbidity after autogenous grafting in which the ramus is used as the donor site [30]. Silva et al. reported that paresthesia was observed in 8.3% of patients after graft removal from the ramus region [31]. Another study reported that, although 18.51% of patients experienced paresthesia in the short term, only one patient still experienced paresthesia during the 12-month follow-up period [16]. In terms of soft tissue injury frequency, the prevalence of inferior alveolar nerve injury in this study was consistent with that reported in the literature. The nerve was exposed in five patients (three involving piezosurgery and two involving conventional surgery); paresthesia lasting up to 6 weeks, but no permanent paresthesia, was observed. The risk of injury to the inferior alveolar nerve during piezosurgery is low; however, it is not absent. It is essential to examine the relationship of the nerve with the cortical bone in detail during preoperative 3D imaging planning; in cases where the nerve courses superior to the incision line, it is essential to leave the incision superficially at the cortical level and apply the chisel carefully. In our study, we did not perform statistical analysis in terms of complications, but we encountered numerically similar complications. We interpreted that the reason for the similar frequency of neurosensory disturbance between the two groups is not due to direct damage to the nerve with the drill but that damage to the nerve may occur during the separation of the bone with the osteotome.

The strengths of this study lie in its design as a randomized control trial, which ensures uniformity in critical group characteristics as well as randomization and patient blinding. Its validity is further strengthened using a well-validated instrument and the fact that all surgeries were performed by a single highly skilled surgeon competent in piezosurgery. Furthermore, the consistent use of cooled saline solution for irrigation in both groups demonstrates methodologic rigor. However, limitations such as the impossibility of double-blinding and the surgeon’s knowledge of group assignments, although inherent in the study, are important considerations in the interpretation of the findings.

The limitations of our study include the inability to implement double blinding and complete masking of the operator, which were inherent constraints of our methodology. Additionally, we did not conduct a statistical analysis to compare complications, treatment costs, and quality of life indicators between the groups. Our focus was primarily on the speed of the two methods, and we did not measure the graft volume, which could be a significant factor in assessing the efficacy of these techniques. The study also did not consistently match the donor and recipient sites ipsilaterally, which might have influenced morbidity-related outcomes. It is essential for future studies to consider the correlation between donor and recipient site locations in evaluating these surgical methods. While our findings align with those observed in other oral and maxillofacial surgery procedures, they should not be generalized across all maxillofacial surgeries. We intend to undertake further research to measure the maximum graft volume obtainable with each technique and to compare the efficiency and postoperative morbidity between them. Comprehensive future studies are necessary to establish equivalency and investigate factors influencing operation time and complication rates. Additionally, the exploration of advanced 3D measurement techniques for assessing postoperative edema and swelling could provide a more nuanced understanding of surgical outcomes [32].

## Conclusions

In conclusion, this study demonstrates that piezosurgery is a safe and effective technique for harvesting autogenous grafts from the ramus region. Furthermore, it offers several advantages without extending the osteotomy or overall operation time, compared to the conventional method, when obtaining grafts from this specific intraoral site.

## Data Availability

The data of the submitted paper are available upon request by e-mail to the corresponding author.
